# The Critical Impact of Sphingolipid Metabolism in Breast Cancer Progression and Drug Response

**DOI:** 10.3390/ijms24032107

**Published:** 2023-01-20

**Authors:** Paola Antonia Corsetto, Stefania Zava, Angela Maria Rizzo, Irma Colombo

**Affiliations:** Department of Pharmacological and Biomolecular Sciences, Università degli Studi di Milano, Via D. Trentacoste 2, 20134 Milano, Italy

**Keywords:** breast cancer, sphingolipids, lipids, metabolism, ceramide, sphingomyelin, drug sensitivity, drug resistance, biomarkers

## Abstract

Breast cancer is the second leading cause of cancer-related death in women in the world, and its management includes a combination of surgery, radiation therapy, chemotherapy, and immunotherapy, whose effectiveness depends largely, but not exclusively, on the molecular subtype (Luminal A, Luminal B, HER2+ and Triple Negative). All breast cancer subtypes are accompanied by peculiar and substantial changes in sphingolipid metabolism. Alterations in sphingolipid metabolite levels, such as ceramides, dihydroceramide, sphingosine, sphingosine-1-phosphate, and sphingomyelin, as well as in their biosynthetic and catabolic enzymatic pathways, have emerged as molecular mechanisms by which breast cancer cells grow, respond to or escape therapeutic interventions and could take on diagnostic and prognostic value. In this review, we summarize the current landscape around two main themes: 1. sphingolipid metabolites, enzymes and transport proteins that have been found dysregulated in human breast cancer cells and/or tissues; 2. sphingolipid-driven mechanisms that allow breast cancer cells to respond to or evade therapies. Having a complete picture of the impact of the sphingolipid metabolism in the development and progression of breast cancer may provide an effective means to improve and personalize treatments and reduce associated drug resistance.

## 1. Introduction

Breast cancer (BC) continues to be the second leading cause of cancer death among women worldwide, despite, over the last 30 years, BC death rates dropping due to increased awareness of the disease, advances in diagnosis, and more effective therapeutic interventions [1]. Major advances regarding diagnosis, prognosis, and treatment of BC, including in surgery, chemotherapy, immunotherapy, and radiotherapy, are due to multiple and interdisciplinary approaches which, over time, have led to a progressive improvement in the classification of BCs [2,3,4,5].

Currently, the classification system of BCs considered most useful and effective, and therefore increasingly used by clinicians for the choice of therapeutic intervention, is based on the presence, or absence, of specific biomarkers in tumor specimens [3,6]. In particular, according to estrogen receptor (ER) and progesterone receptor (PR) expression levels and human epidermal growth factor receptor 2 (ErbB2, also known as HER2) status, BCs are basically divided into four main subtypes [3,6]. Luminal A and Luminal B BCs are both positive for hormone receptors (ER+ and PR+) but differ in the HER2 status (Luminal A is HER2-, whereas Luminal B is HER2+) and in the expression levels of the cell differentiation marker Ki67 (> in Luminal B vs. Luminal A), so that the Luminal A subtype is a low-grade breast tumor, whereas the Luminal B subtype is more aggressive [7]; Luminal B tumors, expressing both hormone receptors and overexpressing HER2, are also named triple positive (TPBC). The HER2-enriched (HER2+) BC subtype overexpresses the oncogene HER2 and includes the HER2+, ER-, and PR- tumors. Finally, the triple negative BC (TNBC) subtype lacks all three biomarkers (ER-, PR-, HER2-) and is characterized by a shorter survival and an early peak of distant recurrence [8].

All four main BC molecular subtypes have also been characterized by significant differences in terms of response to therapy [3,6]. ER+ tumors (roughly 70% of BCs) are usually primarily treated with endocrine therapy, whereas HER2+ BCs (10–15% of BCs) can be firstly managed with monoclonal antibodies that specifically target this receptor. For the remaining 15–20% of BCs, which are ER-/PR-/HER2-, with a more aggressive and metastatic phenotype, increased risk of recurrence, and worse prognosis, a targeted treatment is still missing [8]; as TNBCs do not respond to hormone or antibody therapies, the primary choice for their management is usually chemotherapy, possibly in combination with surgery and radiotherapy. Collectively, many of these therapeutical approaches are demonstrated to improve patients’ survival rates. However, not all BC patients are able to benefit from them as their efficacy may be also limited by both innate and acquired factors, resulting in recurrent cancers often being more resistant to treatment [9].

Therefore, to improve BC management, the main challenges that remain open are the discovery of novel biomarkers with optimal specificity and sensitivity that can be useful in prevention, diagnosis and treatment of the disease; the identification of new molecular targets for the developing of new therapies; and, last but not least, the overcoming of the treatment-resistant relapse.

Today, reprogramming of the cellular metabolism represents a crucial hallmark of BC, and alterations in lipid metabolism, such as de novo lipogenesis, have been shown to be closely related to the decreased sensitivity to traditional chemotherapies and targeted therapies [10]. In the lipid context, in recent decades, many and very appealing interconnections have been also described among BC development, anticancer therapies, drug resistance, and the metabolism of a wide array of bioeffector lipids named sphingolipids (SLs) [11].

**Figure 1:**
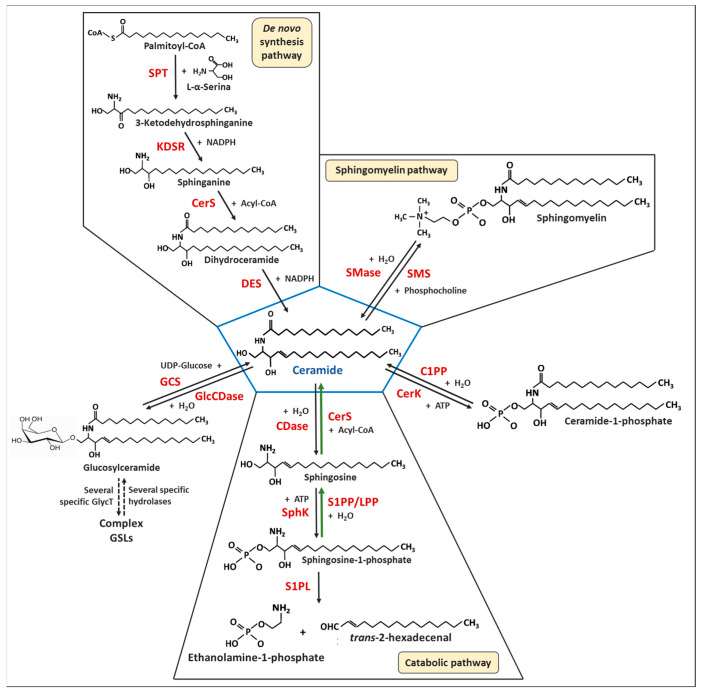
Metabolic pathways of sphingolipids. Abbreviations: CDases, ceramidases; CerK, ceramide kinase; CerS, (dihydro)ceramide synthase; C1PP, ceramide-1-phosphate phosphatase; DES, dihydroceramide desaturase; GCS, glucosylceramide synthase; GlcCDase, glucosylceramidase; GSLs, glycosphingolipids; KDSR, 3-ketosphinganine reductase; LPP, lipid phosphate phosphatases; SMase, sphingomyelinase; SMS, sphingomyelin synthase; SphK, sphingosine kinase; S1PL, sphingosine-1-phosphate lyase; S1PP, sphingosine 1-phosphate phosphatase; SPT, L-serine palmitoyltransferase. Green arrows indicate the salvage pathway.

SLs are a class of chemically heterogeneous amphipathic biomolecules which share a basic sphingoid backbone, mainly represented by the organic aliphatic eighteen carbon amino alcohol sphingosine, whose modifications (including phosphorylation, N-acylation and glycosylation) (Figure 1) through an interlinked metabolic network of enzymes give rise to a broad range of bioactive metabolites with significant roles in membrane biology and a multitude of cellular functions [12,13,14].

Indeed, although SLs were first described as simple structural components of cell membranes, chemical variations in the sphingoid base, N-linked fatty acid and/or polar head group can significantly affect their segregation properties in the cellular membranes [15], so as to influence their fluidity and dynamics [16] as well as the formation, composition and secretion of exosomes that are implicated in cell–cell communication [12,13]. Furthermore, by contributing significantly to the organization of membrane domains and acting both directly, through lipid–protein interactions, and indirectly, through the promotion of physical changes in the membrane itself [15,17], SLs are also key regulators of ion channels and resident receptors so as to have a great impact on membrane permeability and cell signaling [18]. In particular, as intracellular and extracellular mediators, bioactive SLs have been demonstrated to be crucial actors in the modulation of a plethora of different cellular processes that include cell growth and survival, cell differentiation and senescence, angiogenesis, cell adhesion and migration, inflammation, immune response, nutrient uptake, metabolism, responses to stress stimuli and autophagy, and organogenesis [12,13,14]. Although some SLs control these functions within the cell signal transduction networks, dependent on G protein-coupled receptors (GPCRs), protein kinases and protein phosphatases, the mechanisms of action of SLs and their downstream targets remain largely unknown.

As schematically depicted in Figure 1, the central position of SL metabolism is occupied by ceramide (Cer), whose endogenous levels and clearance are regulated by complex and integrated metabolic pathways. Almost all the enzymes involved in both anabolism and catabolism of SLs have been identified and characterized in terms of cell localization and/or substrate specificity [12,19]. Furthermore, a plethora of studies have also ascertained that the abundance of each SL species is strictly controlled by the altered expression and/or activity of these enzymes, so that it is the constant flux of metabolic interconversion of the different SL intermediates that allows them to reach and keep themselves in proper and healthy balance. Indeed, the dysregulation of SL homeostasis can play a critical role in the development and progression of various deleterious pathophysiological conditions, including cancer [11,20,21,22]. In addition, due to their peculiar chemical properties, many SL species often remain within the membranes and compartments where they are produced, with important implications both for metabolism and signaling and for therapeutics, and may demand a more surgical targeting of SL metabolism for the desired outcome to be achieved [23].

SL species that are currently believed to be most widely relevant in BC development and progression, as well as in response to therapy, are Cer and sphingosine-1-phosphate (S1P). Indeed, a lot of studies have demonstrated that the intracellular increase of Cer levels is mainly related to the induction of cell cycle arrest, apoptosis, and autophagy, whereas elevated S1P levels are closely associated with increased cell survival and proliferation, promotion of cell migration and invasion, and prevention of drug-induced apoptosis [24,25,26]. In addition, since S1P is generated from Cer-derived sphingosine (Spn) by the action of sphingosine kinase 1 (SphK1), commonly found upregulated in BCs and related to poorer prognosis and tumor progression, changes in S1P/Cer ratio and SphK1 activity are currently the best characterized outcomes of the alterations of SL metabolism in BCs, along with their effects on chemotherapy and chemoresistance [24,25,26] and on the tumor microenvironment (TME) [26]. Consequently, the inherent increase in Cer levels by suppressing S1P formation and/or accumulation is becoming a promising target for inhibiting tumor growth and overcoming drug resistance. The role and mechanisms of S1P and SphK1 in BCs have been recently and extensively reviewed [24,25,26]. Moreover, in these papers, the authors have also extensively discussed the preclinical studies carried out in both ER+ and TNBC mouse models with inhibitors of SphK1 and other compounds that target the S1P axis, suggesting them as a good promise for reducing BC growth and metastasis. Therefore, we do not further discuss them in this review.

Since other significant variations were described in BC sphingolipidome, both during the multi-step process of breast tumorigenesis and in response to therapy, further nodes in SL metabolic pathways might represent novel therapeutic targets for the development of new anticancer intervention strategies. For example, the specific effects of Cer on cell survival or cell death largely depend on the length of the covalently bound fatty acids [27]. Ceramide-1-phosfate (C1P) can promote cell migration through the activation of the transcription factor NF-kB and the PI3K/Akt and MEK/ERK1-2 pathways by interacting with a receptor coupled to G proteins in the plasma membrane [28], and it can also enhance the expression level of multidrug resistance protein 1/Pglycoprotein (MRP1/Pgp) and breast cancer resistance protein (BCRP) through the up-regulation of prostaglandin E_2_ (PGE_2_) [29]. However, the overexpression of MRP1/Pgp can be also due to glucosylceramide (GlcCer); indeed, this effect can be reversed by blocking UDP-glucose ceramide glycosyltransferase (UGCG or glucosylceramide synthase, GCS) activity [30,31]. Moreover, whereas the hydrolysis of sphingomyelins (SMs) to Cer plays a role in cancer development through the altered expression of SMases, elevated levels of SMs have been associated with the MRP1/Pgp [22].

Therefore, in this review, we will focus on pathways that current research has documented to be impacting both BC development and progression and response to therapies, discussing particularly the functions, characteristics and potential therapeutical relevance of enzymes summarized in Table 1. We hope that this overview will help to highlight the potential of the manipulation of the SL metabolism as a means to increase the efficiency of therapeutic interventions, particularly for TNBC and tumors that show resistance to typical first line treatments.

## 2. Sphingolipid Metabolism—Overview

Sphingolipid (SL) metabolism consists of complex and integrated metabolic pathways in which Cer is the central hub and several intermediates are interconverted in constant and well-orchestrated flux (Figure 1) [12,19]. Indeed, endogenous Cer can be synthetized through the de novo pathway that starts in the endoplasmic reticulum (ER) with the condensation of serine and palmitoyl-CoA by L-serine:palmitoyl-CoA transferase (SPT) to yield 3-ketodehydrosphinganine (3KDS), which is subsequently reduced to sphinganine (Spn, also named dihydrosphingosine) by the action of 3-ketosphinganine reductase (KDSR). Spn is then the substrate for dihydroceramide synthases (more commonly referred to as ceramide synthase, CerS), which N-acylate Spn with a second fatty acid generating dihydroceramide (dhCer). Finally, dhCer is desaturated between C4 and C5 by the action of dihydroceramide desaturase (DES) producing Cer. Cer may be also obtained through the hydrolysis of membrane sphingomyelins (SMs) or glucosylceramide (GlcCer) by the action of sphingomyelinases (SMases) or glucosylceramidases, respectively. Alternatively, Cer can be achieved through the dephosphorylation of ceramide-1-phosphate (C1P) by the action of ceramide-1-phosphate phosphatase (C1PP). Finally, Cer can be produced in the salvage pathway, which primarily utilizes the recycling of sphingosine (Sph), a product of SL catabolism. Note that, whereas de novo biosynthesis pathway produces Spn, the salvage pathway generates Sph; these two sphingoid bases differ structurally in the trans-double bond located between C4 and C5 (present in Sph, but not in Spn), but both can be used as a substrate by CerS [12,19]. 

Once produced, Cer can partially accumulate or be converted into other different SL species. It can be phosphorylated by ceramide kinase (CerK) to form ceramide-1-phosphate (C1P), or it can be glycosylated by glucosylceramide synthase (GCS) to produce GlcCer from which other, more complex GSLs can be originated. Cer may be also reversed into SM by the addition of a phosphocholine group by the action of sphingomyelin synthase (SMS). Finally, Cer can also be degraded by ceramidases (CDases) to produce Sph, which may be recycled into Cer through the salvage path or phosphorylated by sphingosine kinase (SphK) to form sphingosine-1-phosphate (S1P). S1P, in turn, may regenerate Spn by dephosphorylation catalyzed by sphingosine-1-phosphate phosphatase (Sph1PP) or permanently exit the SL metabolic pathway by the action of sphingosine-1-phosphate lyase (S1PL1), which releases ethanolamine-1-phosphate and hexadecenal [12,19].

**Figure 2:**
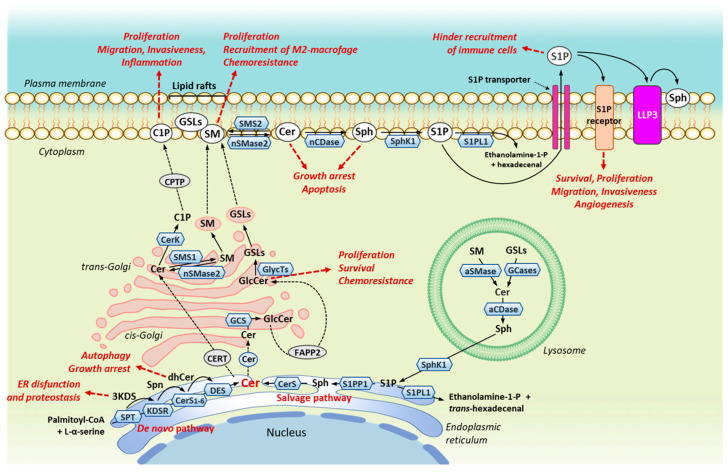
Cellular localization of sphingolipid metabolic enzymes primarily altered in breast cancer and biological effects on tumor cells. Abbreviations: aCDase, acid ceramidase; aSMase, acid sphingomyelinase; Cer, ceramide; CerK, ceramide kinase; CerS1-6, (dihydro)ceramide synthase 1-6; CERT, ceramide transfer protein; C1P, ceramide-1-phosphate; CPTP, ceramide phosphate transfer protein; DES, dihydroceramide desaturase; dhCer, dihydroceramide; FAPP2, phosphatidylinositol-4-phosphate adaptor protein 2; GCS, glucosylceramide synthase; GlcCer, glucosylceramide; GSLs, glycosphingolipids; 3KDS, 3-ketosphinganine; KDSR, 3-ketosphinganine reductase; LPP3, lipid phosphate phosphatase 3; nCDase, neutral ceramidase; nSMase2, neutral sphingomyelinase 2; SMS1/2, sphingomyelin synthase 1/2; S1P, sphingosine-1-phosphate; Sph, sphingosine; SphK1, sphingosine kinase 1; Spn, sphinganine; S1PL1, sphingosine-1-phosphate lyase; S1PP1, sphingosine-1-phosphate phosphatase 1; SPT, L-serine palmitoyltransferase.

Dysregulation of the SL homeostasis, due to alterations in the activity of several enzymes involved in SL metabolism, may affect BC cell survival and death, as well as modulate responses of BCs to chemotherapy [20,21,80]. In addition, accumulating evidence indicates that these effects are breast tumor specific, and that distinct SL species, which are generated in different subcellular compartments and/or by different metabolic pathways (Figure 2), may have different functions and/or different mechanisms of action in mediating the survival or death of BC cells [21,22,81].

## 3. Alterations in Ceramide De Novo Biosynthesis Enzymes in Breast Cancer Growth and Drug Response

### 3.1. Serine Palmitoyl-CoA Transferase (SPT) and 3-Ketosphinganine Reductase (KSDR)

SPT is a trimeric enzyme localized in the ER (Figure 2), which catalyzes the critical rate-limiting step of the de novo biosynthetic pathway of Cer, producing the long-chain (sphingoid) base (LCB) (Figure 1) [32]. The relevance of LCB length and structure in inducing apoptosis in MCF-7 BC cells [33], in endocrine-resistant MDA-MB-231 cells, in chemoresistant MCF-7/TN-R [34], and in MCF-7/adriamycin (MCF-7/Adr) drug-resistant cells [35] was demonstrated using various Cer analogues. However, LCB variants can also be originated by changes in the amino acid preference substrate due to specific mutations in one of the two primary subunits of SPT [82,83]. In particular, the use of L-alanine or L-glycine as alternative substrates to L-serine yields the production of non-canonical LCBs, such as 1-deoxySpn and 1-deoxymethylSpn, which cannot be further modified and form Cer due to a lack of the hydroxyl group in C1 [14] and can accumulate to the point where they can induce neurotoxicity, a side effect also observed in paclitaxel-treated BC patients (Table 1) [32]. Since elevated SPT-mediated 1-deoxysphingoid levels were detected in serum samples from BC patients who received paclitaxel treatment, and a major dose-limiting toxicity for treatment with this drug has been shown [84], it has been suggested that the induction of the metabolic switch from SPT-dependent generation of 1-deoxysphingoid bases to conventional Spn by the use of L-serine can be a potential therapeutic intervention to alleviate paclitaxel-induced neurotoxicity [20,84].

Furthermore, Spears et al. [36] have recently reported that SPT is upregulated in several types of cancer cells, including the TNBC MDA-MB-231 cells. Since 3KDS is toxic and tumor cells depend on KDSR activity to maintain 3KDS homeostasis, these authors have suggested that the overexpression of SPT can be exploited to selectively poison cancer cells and KDSR was indicated as a potential therapeutic target for the development of specific inhibitors. Indeed, in cancer cells, but not in normal cells, the inhibition of KDSR induces the toxic accumulation of 3KDS, leading to ER dysfunction and loss of proteostasis (Table 1) [36].

### 3.2. Ceramide Synthase (CerS)

Until now, six different isoforms of CerS, named CerS1-6, have been identified. All the CerS are integral membrane proteins mainly localized in the ER (Figure 2), and are responsible for the attachment of an acyl group from a fatty acyl-CoA to the free ammino group of Spn producing dhCer (Figure 1). However, each CerS differs from the other in its tissue distribution and specific preference for the length of the fatty acyl-CoA used for N-acylation [12,27]. CerS1 is strongly expressed in the brain and skeletal muscle, and selectively prefers C18:0- and C18:1-CoA. Cers2 is the most abundantly and ubiquitously expressed isoform in mammalian tissues and has a substrate specificity towards C20:0-, C22:0-, C24:0-, C24:1-and C26:0-CoA. CerS3 is primarily detected in the testis, and preferentially uses very long -chain-CoA (C26-C32 and higher, possibly also containing 4-6 double bonds). CerS4 is strongly expressed in the skin, heart, and spleen and prefers C18:0-, C20:0- and C22:0-CoA. Finally, CerS5 and CerS6, which largely differ in tissue distribution, primarily uses C14:0- and C16:0-CoA [12]. 

The discovery of CerS1-6 has represented a key element in focusing on the opposite roles that Cer with different fatty acyl chain lengths could play in BC development, drug sensitivity and/or drug resistance (Table 1). Indeed, higher levels of CerS6, producing mainly C14:0- and C16:0-Cer, were detected in human malignant BCs compared with corresponding healthy tissues and were associated with poor prognosis [40,41,42], lymph node involvement and metastasis [42]. Higher CerS6 levels were also observed in ER+ BCs with respect to ER- BCs [40,43,44], in Luminal BCs with respect to TNBCs and in epithelial-like cancer cells with respect to mesenchymal-like cancer cells [45], suggesting that the downregulation of CerS6 observed in the epithelial mesenchymal transition (EMT) might have a pivotal role in the regulation of cell migration. In particular, whereas the CerS6 overexpression, which significantly increased the C16:0-Cer content, was capable of inhibiting the cell migration of two mesenchymal human BC cells (i.e., MDA-MB-231 and MDA-MB-468 cells) by the reduction of the plasma membrane fluidity, the CerS6 downregulation in two epithelial tumor cells (i.e., MCF-7 and T47D cells), which was associated with the reduction of C16:0-Cer, increased basal and CD95-mediated plasma membrane fluidity and cell migration [45]. Interestingly, Edmond’s group has also demonstrated that the reduction of the plasma membrane fluidity and cell migration of MDA-MB-231 and MDA-MB-468 cells could be also obtained by treating cells with non-cytotoxic concentrations of exogenous C16:0-Cer [45], predicting that in women with aggressive TNBCs where CerS6 is not expressed, a slight increase in the level of C16:0-Cer might help to reduce their elevated risk of metastatic dissemination. However, a possible exogenous treatment with Cer with long-chain fatty acids presents problems for their delivery as chemotherapeutic agents in vivo due to decreased solubility and bioavailability. To overcome these problems, C16:0- or C18:0-pyridinium Cer have been synthesized with increased water solubility and cell membrane permeability [85,86], and liposome-mediated delivery of C6-Cer was able to induce apoptosis in MDA-MB-231 cells by inhibiting phosphorylated Akt levels and stimulating caspase-3/7 activity more effectively than nonliposomal ceramide [87].

The discovery of the dramatical upregulation of the long noncoding RNA (lncRNA) CERS6-AS1 in BC tissues compared to that in continuous normal tissues, its close positive correlation with the tumor differentiation grade, TNM stage, and poor prognosis, as well as the remarkable increase of CERS6-AS1 related to elevated CerS6 levels, cell migration and invasion in BC cells (i.e., MDA-MB-231, MDA-MB-436, MDA-MB-453 and MCF-7 cells) with respect to normal human breast cells (i.e., MCF-10A), with the lowest expression in MDA-MB-231 cells and the highest in MCF-7 cells, have led to attribute a role to this lncRNA in the promotion of CerS6-dependent malignancy in BCs and to provide a novel therapeutic target for BC to improve prognosis [88]. This study has also demonstrated that CERS6-AS1 was capable of accelerating BC cell proliferation and suppressing BC cell apoptosis by promoting CerS6 mRNA stability through the RNA binding protein IGF2BP3 [88]. The relevance of CERS6-AS1 as an oncogene in BCs has been subsequently confirmed in a nude mouse model xenografted with MDA-MB-231 cells overexpressing CERS6-AS1 by Yan et al. [89], who further documented that CERS6-AS1 can promote cell proliferation and inhibit cell apoptosis by downregulating the expression of the tumor suppressor microRNA-125a-5p, which exhibits opposite effects to those of lncRNA CERS6-AS1, and by upregulating the BC susceptibility to gene 1-associated protein 1 (BAP1). These results further support the view that the CERS6-AS1/microRNA-125a-5p/BAP1 might serve as a potential therapeutic target for BCs [89].

CerS6 targeting could also provide a therapeutic option to improve the sensitivity of BC cells to death receptor signaling and apoptosis. Indeed, the knock-down of CerS6 and depletion of its product C16:0-Cer have been identified as upstream effectors of the loss of focal adhesion protein and plasma membrane permeabilization, via the activation of caspase-7, in TNF-α treated MCF-7 cells [46]. Finally, CerS6 could also play a positive role in drug-induced lethal autophagy in some BC cells. Indeed, the combined treatment of TPBC cells (BT-474 cells) with the antifolate pemetrexed, the multi-kinase inhibitor sorafenib and/or FTY720 resulted in CerS6-dependent toxic autophagy that increased cytotoxicity of pemetrexed [47,90]. Mechanistically, CerS6 could improve the autophagic process and pemetrexed-induced cell death by the activation of PP2A which, in turn, via SRC and ERK kinase signaling activation, induces cell death through the formation of autophagic vesicles and acidic endosomes [47].

Contrary to CerS6, elevated expression of CerS2, the only CerS that synthesizes Cer with very long acyl chains (C22:0, C24:0, C24:1), has been found in a strong positive correlation with the longer disease-free times and overall survival of the patients with respect to those with CerS2 negative tumors, and in a reverse relationship with tumor progression, lymph node metastasis and HER2 expression [37,38]. The Fun’s group has also demonstrated the involvement of CerS2 in chemotherapeutic outcomes both in vitro and in vivo, suggesting that low CerS2 expression may predict chemoresistance. Indeed, whereas CerS2 expression was significantly lower in drug-resistant MCF-7/Adr cells compared to the drug-sensitive MCF-7 cells, the overexpression of Cers2 in MCF-7/Adr cells increased the chemosensitivity to multiple chemotherapeutic agents, including doxorubicin (Dox); on the contrary, the susceptibility to Dox cytotoxicity was reduced in MCF-7 cells after CerS2 knockdown [37]. Furthermore, the combination of CerS2 overexpression and Dox significantly inhibited the growth of xenografts in nude mice [37]. Higher CerS2 mRNA and protein levels were also found in poorly invasive Luminal A MCF-7 cells compared to highly invasive TNBC MDA-231-MB cells: whereas CerS2 overexpression in TNBC cells significantly inhibited their migration and invasion ability, the opposite effects (significant increase in migration and invasion) were observed in MCF-7 cells after CerS2 knockdown [39]. In addition, the CerS2-mediated inhibition of cell invasion was associated with decreased vacuolar H^+^-ATPase (V-ATPase) activity and extracellular hydrogen ion concentration and, consequently, with the activation of secreted MMP-2/MMP-9 which ultimately suppressed the tumor’s invasion by the degradation of the extracellular matrix. Then, CerS2 may represent, as suggested by the authors, a novel target for selectively disrupting V-ATPase activity and the invasive potential of TNBCs [39]. Furthermore, since V-ATPase acidifies endosomal-lysosomal organelles and the microenvironment of tumor cells, promoting not only tumor invasion and metastasis but also chemoresistance [91], the elevated lysosomal and extracellular pH due to the inhibition of V-ATPase by CerS2 in Dox-treated MCF-7/Adr cells could be the mechanism which facilitates cellular entry and nuclear localization of Dox [37]. Finally, although up to now no correlation has been found between the significant lower CerS3 mRNA levels detected in BC specimens compared to normal breast tissues and patients’ survival [42], it has been reported that the complete block of cellular synthesis of C22:0- and C24:0-Cer in TPBC BT-474 cells by simultaneous knockdown of CerS2 and CerS3 enhances cell death through the induction of lethal autophagy upon the combined exposure to the anti-folate pemetrexed and FTY720 [90], supporting further the protective role of very long chain-Cer in BC chemosensitivity and indicating CerS3 as a further potentially useful target to overcome drug resistance [27].

However, conflicting data are published for CerS2 expression in BCs. A positive correlation between elevated C24:0- and C24:1-Cer levels and increased CerS2 mRNA expression in malignant BCs compared to benign BCs was reported by Schiffmann et al. [40], suggesting a particularly ambiguous role of CerS2 in terms of induction of malignancy. Increased CerS2 mRNA expression in human BC tissues in comparison to continuous healthy tissues was also documented by two other studies [41,42]. However, in these studies the elevated levels in cancer specimens compared to normal tissues were consistent throughout all Cer species (C14:0, C16:0, C18:0, C18:1, C20:0, C22:0, C24:0, C24:1, C26:0 and C26:1), due to a significant increase of activity of CerS2, CerS4, Cer5 and CerS6, suggesting that the effects of the different Cer species on BC growth might result from complex and inter-regulated activity of different CerS. In line with this assumption, there is a subset of patients who exhibited a positive correlation between the mRNA levels of CerS4 and CerS2, and between CerS4, CerS2 and CerS6 [41]. The upregulation of CerS5 mRNA in response to the selective knockdown of either CerS2 or CerS6, and vice versa, were also observed in MCF-7 cells [91,92]. In particular, through the manipulation of cellular Cer levels by overexpression, CerS 2, 4 or 6 in MCF-7 cells Hartmann’s group [92] highlighted that very long chain-Cer produced by CerS2 must be in equilibrium with long chain-Cer generated by CerS4 and CerS6 for normal cell growth, whereas the increase of CerS2, CerS4 and CerS6 detected in BC tissues and cells might be the physiological reaction of the tumor aimed at counteracting an imbalance between Cer species that promote and inhibit tumor cell growth.

Although it cannot be excluded that the concerted activity of the different CerS isoforms may be influenced by the formation of heterodimers of CerS family members [93], a recent study has shown that the qualitative and quantitative expression of different CerS2 transcripts produced via alternative splicing (AS) could explain the contrasting effects (pro- and anti-proliferative, pro- and anti-apoptotic) of very long chain-Cer and then play a crucial role in BC pathogenesis [94]. Indeed, higher levels of the alternative spliced transcript for CerS2 (AS1 transcript), lacking exon 8, which confers catalytic activity and assists in substrate binding to synthesize very long chain-Cer, were found in Luminal B tumor tissues as compared to normal tissues, as well as in TPBC BT-474 cells as compared to normal MCF-7A cells, whereas the expression of normal CerS2 transcript (PC transcript) was not markedly different. Since elevated AS1 transcript expression caused a drastic reduction of very long chain Cer levels, despite the presence of PC transcript at normal levels, it was concluded that it is the overexpression of AS1 transcript that allows the increased proliferation and migration of Luminal B cancer cells, leading to a poor prognosis outcome for the survival of patients with this subtype of BC [94]. In addition, assuming that the differential expression of alternative transcripts of the other CerS could provide a unique specific signature for each BC subtype and be responsible for the accumulation of chain length-specific Cer, to explain the contrasting data of their pro- or anti-tumoral effects these authors analyzed alternative splicing events of CerS genes for each molecular BC subtype, finding that, whereas an AS event in CerS2 is unique in Luminal B subtype, an AS event in CerS4 is specific for the TNBC subtype, and suggesting specific AS events in CerS genes as a regulatory mechanism that deregulates Cer expression in BCs and can be explored further as a suitable therapeutic target [94].

Collectively, this evidence confirms that not all Cer containing different fatty acid chain lengths play similar roles in BC development, cell death and survival, and response to therapy (Table 1), although the role of different CerS is far from being completely understood because the expression of various CerS genes may also depend on the type, stage and grade of BCs, and the levels of each Cer species can also result from the complex and finely inter-regulated activity of different CerS. In any case, the identification of the molecular mechanisms underlying how these Cer exert their distinct effects based on fatty acid composition remains a crucial node for generating novel therapeutics centered on SL metabolism modulation.

### 3.3. Dihydroceramide Desaturase (DES)

DES is the enzyme localized in RE responsible for inserting the 4,5-trans-double bond into the LCB of dhCer produced by CerS1-6 to generate Cer (Figure 1 and Figure 2). Two isoforms of DES have been identified: DES2 has a dual function due to desaturase and hydroxylase and is preferentially expressed in the kidney, small intestine, and skin, where the generation of phytoceramides is essential. On the other hand, DES1 is ubiquitously expressed with high DES activity and very low hydroxylase activity [95].

Although dhCers have long been considered a biologically inactive molecule, evidence is now available to justify the relevance for these SL species in BC as well. Indeed, fenretinide [N-(4-hydroxyphenil)retinamide; 4-HPR], a synthetic derivative of all-trans retinoic acid characterized by high cytotoxic efficacy against cancer cells in vitro, was the first chemotherapeutic drug found to inhibit DES1, resulting in a reduction of the Cer de novo biosynthesis and the accumulation of endogenous dhCers [96]. In addition, although the results were not exciting, the role of DES1 in the development and growth of BCs and in preventing relapses is supported using 4-HPR, alone or in combination with tamoxifen, in several clinical trials (Table 1) [49]. As the disappointing in vivo activity of 4-HPR could be due to plasma drug concentrations remaining below the minimum threshold for the onset of antitumor activity, and also after multiple and prolonged administrations because of the poor solubility in water and consequent low bioavailability of the drug, a new 4-HPR formulation (referred as nanofenretinide, NanoFEN), characterized by improved aqueous solubility through drug salification and complexation with 2-hydroxypropyl β-cyclodextrin (a solubilizing excipient endowed with favorable biodistribution and reduced toxicity), has been produced [50]. NanoFEN was showed to produce a massive accumulation of bioactive dhCer associated, and high, durable anticancer efficacy both in vitro and in vivo against cell lines from multiple tumors, including MCF-7 and MDA-MB-231 cell lines, and tumor xenografts. The absence of macroscopic toxic effects and the activation of multiple signaling pathways leading to apoptosis, autophagy, and proliferative/metabolic inhibition strongly support its future use in clinical trials [50].

Recently, Linzer et al. [48] have also showed that increased DES1 levels, found in a subset of HER2+ tumors, were associated with the more aggressive and metastatic phenotype of this BC subtype and with worse survival outcomes of the patients (Table 1). In addition, as the suppression of dhCer levels by 4-HPR was associated with HER2-driven anchorage-independent survival (AIS) in HER2+ SKBR3 cell lines and the DES1 expression was necessary and sufficient to acquire and drive AIS in breast epithelial cells so to increase tumorigenicity in HER2+ BC cells, it has been concluded that DES1 may have utility as a biomarker of aggressive and metastasis-prone HER2+ BC and targeting DES1 may be an effective approach for overcoming AIS. Mechanistically, it has been also demonstrated that DES1 is not only regulated at the post-translational level by HER2, but is also a key downstream effector of HER2-PI3K signaling driven glucose uptake and metabolism [48].

Altogether, this evidence supports the view that complex and distinct mechanisms, mainly involving CerS1-6 and/or DES1 enzymes, can control de novo generated Cer levels in BC cells (Table 1) and support further efforts to confirm CerS and/or DES1 as potential therapeutic targeting.

## 4. Alterations in Sphingomyelin Metabolism in Breast Cancer Growth and Drug Response

Sphingomyelin (SM) is one of the most abundant SL in biological membranes, where it plays important structural roles by participating in the membrane stability, and it is involved in the regulation of numerous signaling pathways. Indeed, in association with cholesterol and other SLs, SM defines ordered microdomains, which selectively cluster many proteins, including membrane receptors such as epidermal growth factor receptor (EGFR) and transforming growth factor β receptor 1 (TβR1).

Cellular levels of SM are regulated by two families of enzymes: sphingomyelin synthases (SMSs), which convert Cer into SM through the attachment of a polar headgroup to the hydroxyl at the 1-position of Cer, and sphingomyelinases (SMases), which catalyze the hydrolysis of SM, regenerating Cer and releasing the polar headgroup (Figure 1). Although there are three different isoforms of SMs, named SMS1, SMS2 and SMS-related protein (SMSr), only SMS1 and SMS2 promote the transfer of a phosphocholine group from phosphatidylcholine to the Cer generating SM [14], whereas SMSr catalyzes the synthesis of the SM analogue with phosphoethanolamine as the polar headgroup linked to C1 [97]. Furthermore, whereas SMSr localizes in the ER lume, SMS1 is in the trans-Golgi apparatus and SMS2 in the plasma membrane (Figure 2) [98].

SMases also exist as three major groups, depending on the pH required for optimal activity: acid (aSMases), neutral (nSMases) and alkaline (Alk-SMases); whereas aSMSs can be trafficked into either lysosomal (L-aSMase) or secretory (S-aSMase) pathways, nSMase2, which is the most common of the four isoforms of nSMases identified in mammalians, localizes in both Golgi and plasma membrane (Figure 2). Finally, alk-SMases are found only in the liver and gut, where they are a potential prognostic and diagnostic marker of inflammatory bowel disease and colorectal cancer [98].

The prognostic value of SM and its catabolic and anabolic enzymes in BCs have been recently evaluated by Moro et al. [42]. They found that total levels of SM, as well as all SM species containing C14:0-, C 16:0-, C18:0-, C18:1-, C20:0-, C22:0-, C24:0-, C24:1-, C26:0-, and C26:1-CoA, were significantly higher in cancer tissues than in normal breast tissues and were correlated with higher levels of nSMase2 and lower levels of SMS2, thus contributing to the increase of Cer levels, in turn associated with less aggressivity of cancer (Table 1). The role of SMS2 in promoting the aggressive phenotype in BC due to the disruption of the homeostasis between Cer and SM was also substantiated by Zheng et al. [52]. These authors, who in a primary study demonstrated the association between elevated SMS2 expression and BC metastasis, subsequently reported that SMS2 promotes cancer cell proliferation by suppressing a Cer-associated apoptotic pathway, and cancer cell migration and invasiveness by enhancing EMT through the activation of TGF-β/Smad signalling pathway, in both MCF-7 and MDA-MB-231 cell lines and in vivo. Furthermore, SMS2 was able to activate the TGF-β/Smad signaling pathway primarily by increasing TGF-β1 secretion, likely because of the aberrant expression of SM [52]. An interrelation among SM levels, EMT, TGF-β/Smad pathway and an aggressive phenotype of MDA-MB-231 cell line was also observed by Liu et al. [53]. However, these authors reported that the EMT mediated by TGF-β/Smad pathway was negatively regulated by SMS1. Furthermore, when BC cells were treated with TGF-β1, overexpression of SMS1 downregulated TβR1 expression on the cell membrane, possibly due to increased endocytosis, and was able to inhibit MDA-MB-231 cell migration and invasion induced by TGF-β1 [53]. On the contrary, SMS2 expression was neither altered nor correlated with that of TβR1. Collectively, these results support the assumption that both SMS isoforms might have a critical but opposite role in promoting tumor cell migration and invasion by acting on EMT mediated by TGF-β/Smad signaling pathway, and both could provide new insight into the mechanisms underlying BC metastasis. However, a recent study, which confirmed the positive relation between the high SMS2 expression and worse prognosis, and higher SMS2 levels in TNBCs than in Luminal BCs, has shown that SMS2 facilitated M2 macrophage polarization in TNBC cells and that SMS2 inhibition was associated with a higher density of M2-polarized macrophage infiltration in Luminal BCs than TNBCs. Indeed, SMS2 knockout or SMS2 enzyme inhibition can effectively attenuate this effect [51]. Furthermore, SMS2 was able to hinder the infiltration of M2-polarized macrophage into the tumor stroma and reduce tumor progression in a mouse model of TNBC [51]. 

The action of SMases has been found to be an essential step for the efficacy of chemotherapy and radiotherapy in different types of cancers, and many studies have also linked these enzymes with drug resistance (Table 1) [54]. On the other hand, evidence of their involvement in BC is still limited. A pivotal role of nSMase2 has been reported in daunorubicin (DA)-induced cell death in MCF-7 cells [55]. In particular, DA treatment was able to induce a significant accumulation of the pro-apoptotic Cer with C16:0, C18:0 and C24:0, and a remarkable increase in nSMase2 transcript, with the consequent increment of protein levels and activity, whereas little changes were observed in nSMase1 and nSMase3 mRNAs and only a slight increase in aSMase mRNA. A remarkable upregulation of nSMase2 transcript, associated with the drastic downregulation of SMS2 and SMS3, was also found in Dox-resistant MCF-7 cells compared to Dox-sensitive parental cells, indicating that the SL metabolism in Dox-resistant cells was oriented toward the reduction of Cer levels and concomitant increase in SM, and that Dox-resistant cells tend to escape from Cer-related apoptosis by the activation of SM synthesis [64]. Finally, Lee and Kolesnick [22] have also postulated that increased SM content, developing in some BCs as well as in lymph node metastases, could recruit and functionalize the ATP Binding Cassette Subfamily B Member 1 (ABCB1) in the plasma membrane, imparting the cell to a partial MDR state, which is complemented by the alteration in the composition of GSL-enriched microdomains (GEMs), especially an accumulation of GlcCer and neutral GSLs.

Although the potential role of SM and its metabolizing enzymes in BCs remains to be further elucidated, hopeful proofs have been also obtained considering that SM synthesis in the Golgi is regulated by Cer transport from the ER to the Golgi by ceramide transfer protein (CERT) via a non-vesicular mechanism (Figure 2) [99]. Indeed, the loss of CERT expression could provide an advantage to TNBC cell proliferation through the upregulation of epidermal growth factor receptor (EGFR) signaling, resulting from changes in SM levels at the plasma membrane [100]. Moreover, two studies produced evidence that CERT knockdown was able to sensitize HER2+ BC cells to Dox- or paclitaxel-induced cell death through the induction of Cer-mediated ER stress or lysosome-associated membrane glycoprotein 2 (LAMP2)-dependent autophagic flux [101,102], and a study indicated CERT as a useful predictor of response to neoadjuvant paclitaxel for primary TNBC [103].

Therefore, these results support the view that changes in SM levels, possibly also due to altered Cer trafficking by CERT, might suggest novel strategies for inducing BC cell death or attenuating drug resistance, and that CERT could be a new pharmacological target in cancer resistant to chemotherapy.

## 5. Other Altered Sphingolipid Metabolism Enzymes Involved in Breast Cancer Growth and Drug Resistance

### 5.1. Ceramide Kinase (CerK)

CerK, typically localized in the trans-Golgi (Figure 2), though also found in the plasma membrane, catalyzes the phosphorylation of Cer (preferentially Cer species with acyl chain lengths longer than 12 carbons [14]) to form C1P (Figure 1). Following its production, C1P is transported to the plasma membrane by the ceramide phosphate transfer protein (CPTP) (Figure 2) [104].

The role of CerK in BCs is ascertained by several studies that documented both an overexpression of this enzyme in more than 2000 patients with different BCs subtypes and clinical pathological characteristics and its correlation with tumor aggressivity and poor clinical outcome following adjuvant or neoadjuvant therapy (Table 1) [56,57]. In addition, a gene expression analysis of CerK demonstrated that mRNA levels of this enzyme were higher in ER- BCs than those ER+, and were associated with worst prognosis, shorter survival, and increased risk of recurrence, likely due to the rapid upregulation of CerK following chemotherapy of ER- BCs [58,59]. Furthermore, the Cerk inhibitor NVP-231 was able to reduce MCF-7 cell proliferation by inducing cell cycle arrest and subsequent apoptosis [57], and the overexpression of CerK contributed, to a different extent, to the migration and invasion in both non-metastatic MCF-7 and metastatic MDA-MB-231 cells through PI3K/Akt/Rho kinase activation [60]. These data demonstrated for the first time that CerK promotes migration and invasion of metastatic BC cells and that targeting of CerK had the potential to counteract metastasis in BCs.

The different roles played by CerK in more aggressive metastatic BC cell lines with respect to non-metastatic cell lines have been substantiated in a recent and exhaustive study, reporting a detailed analysis of SL profiles and the transcriptional and translational expressions of corresponding metabolizing enzymes in TPBC BT-474 and TNBC MDA-MB-231 cells: each cell type exhibited a unique SL profile, likely responsible for their unique phenotypic behavior, and common enzymes, including CerK, were dysregulated in both cell lines [61]. However, although CerK can be a potential target to contrast tumor cell proliferation and migration in both cell subtypes, siRNA- or NVP-231-mediated CerK inhibition showed that CerK could play different roles in more aggressive metastatic BC cell lines with respect to non-metastatic cell lines. Indeed, CerK inhibition promoted a striking decrease in Luminal specific BT-474 cell proliferation and a significant tumor regression in the corresponding xenograft model, sustaining the idea that CerK can potentially be a good therapeutic target for primary tumors of Luminal subtype. On the other hand, CerK inhibition in MDA-MB-231 cells was able to induce a significant decrease in cell migration and attenuate the tumor growth in the xenograft tumor model, implying that CerK downregulation was equally important for primary tumor abrogation and the migration of disseminated tumor cells in TNBCs [61].

A positive correlation between high levels of CerK mRNA and its product C1P with Ki67 index in BC tissues, compared to adjacent normal tissues, has been also reported by Bhadwal et al. [105]. In addition, although C1P comprised only 10% of all SL species identified in BC specimens, higher C1P levels in tumor tissues than in normal tissues have suggested C1P as the SL species with a fair predictive ability in discriminating BC tissues and indicated C1P as a potential predictive biomarker in BC. However, no significant relationship was found between C1P/CerK and clinical features of BCs, except for Ki67 levels which were correlated to C1P levels in tumors, but not in normal tissues. A surprising finding from this study was the detection for the first time of Cer and C1P containing fatty acids with a rare, odd chain (i.e., C23:0 and 23:1) in patients’ tissues [105]. Whether odd carbon chain fatty acids are present in other SL species and what their role in BCs may be remain to be determined. 

### 5.2. Glucosylceramide Synthase (GCS)

GCS is the enzyme that localizes in cis-Golgi apparatus (Figure 2) and catalyzes the transfer of one glucose molecule from activated UDP-glucose to the hydroxyl group at C1 of the ceramide in β-linkage (O-linked glycosylation), thus generating GlcCer (Figure 1).

GCS overexpression was reported in BC patients and was related to increased cellular proliferation, tumor progression and poor prognosis [62]. In addition, as GCS mRNA levels were most elevated in late-stage BCs, GCS expression has been suggested as a potentially useful marker of disease progression. However, high GCS mRNA levels were also found in BC patients with ER+ status, lower histological grading, low Ki67 levels and ErbB2 negativity (Table 1) [63].

A crucial role for GCS in the establishment of the multidrug-resistant phenotype in BC cells is supported by several pieces of evidence. Indeed, Che et al. [65] demonstrated that pro-apoptotic Cer levels were suppressed by chemotherapy via increasing mRNA and protein levels of GCS. Furthermore, alterations of several cellular properties, which enhanced proliferation and Dox-resistance, were also described in GCS overexpressing MCF-7 cells [64,66]. Importantly, these cellular effects were mediated by an altered composition of GEMs, especially an accumulation of GlcCer and neutral GSLs, which resulted in an increased gene expression of the ABCB1 [67]. The relevance of ABCB1 was also confirmed by Liu et al. [68], who demonstrated that the silencing of the gene encoding GCG, through the promoter CpG island methylation, was able to downregulate ABCB1 expression. In addition, this downregulation was inversely correlated with drug resistance in ductal BC cells, and sensitized multidrug-resistant cells to chemotherapy through Src and Akt-mediated β-catenin signaling [66,67]. Furthermore, Zhang et al. [69] reported that ABCB1 upregulation in MCF-7 cells could increase the GCS expression with a decrease in Cer levels and cellular apoptosis, whereas ABCB1 downregulation in MCF7/Adr cells results in opposite effects. Then, the decrease and increase of GCS and Cer, respectively, were correlated to increased apoptosis.

In any case, collectively, this evidence supports the view that inhibitors of GCS might be useful in preventing resistance to chemotherapy. Indeed, the GlcCer synthase inhibitor 1-phenyl-2-decanoylamino-3-morpholino-1-propanol (PDMP) was able to restore the sensitivity of BC cells to vinblastine treatment [31] and stimulate autophagy in MCF-7 cells [70], whereas combinations of inhibitors of GCS with 4-HPR, which, as previously reported, is known to elevate Cer and dhCer levels, were reported to synergistically suppress the growth of various human cancer cells, including BCs [106].

Since ABCB1 might also be responsible for the transfer of GlcCer to the lumen of the Golgi apparatus, where LacCer and neutral complex GSLs are produced [107], Morad and Cabot suggested that the drug resistance to tamoxifen related to ABCB1 overexpression could be also due to the increment of Cer clearance following GlcCer accumulation [108]. However, other studies have postulated that GlcCer may regulate its own entrance to the Golgi apparatus in dependence of the length of their acyl-chain, and that the activity of ABCB1 could be not necessary [107], suggesting that there may be an alternative mechanism to regulate the fate of GlcCer. Since the transport of GlcCer into Golgi for the synthesis of LacCer, and then of neutral GSLs, which determine the lipid composition of the plasma membrane [99], is mainly mediated by phosphatidylinositol-4-phosphate adaptor protein 2 (FAPP2), a potential new strategy for inducing BC cell death might be targeting FAPP2 and hindering the synthesis of neutral GLSs. Indeed, Tritz et al. [109] showed that FAPP2 downregulation increases tumor cell sensitivity to Fas-induced apoptosis in metastatic breast MDA-MB-231 cell lines, as well as showing that evidence exists that the invasive properties of MCF-7 are highly correlated with the globo-series GSLs SEEA3 and SSEA4, clustered in the GEMs, and the subsequent activation of focal adhesion kinase (FAK) in GEMs [110,111], whose levels were significantly up-regulated in TNBC and metastatic BC tissues and related to cancer recurrence [112]. In a more recent study, it has also been demonstrated that elevated levels of SSEA3 were significantly correlated with tumor progression and poor survival in patients, and that the globo-series GSLs in BC cells (MCF-7 and MDA-MB-231 cells) form complex membrane lipid rafts with caveolin-1 (CAV1) and focal adhesion kinase (FAK), which in turn interacts with AKT and receptor-interacting protein kinase (RIP), respectively [113]. In addition, the disruption of the complex induced apoptosis though the trigger of the Fas dependent pathway provided not only a link between neutral GLSs and the FAK/CAV1/AKT/RIP complex in tumor progression and apoptosis, but also suggested that hindering the formation of neutral GLSS might represent a new direction for the treatment of BC. However, further studies are required to establish the connection of GCS with ABCB1 and FAPP2.

### 5.3. Ceramidases (CDases)

CDases are the enzymes responsible for the breakdown of Cer to produce a free fatty acid and Sph, which can be either recycled through the salvage pathway or phosphorylated by SphK1/2 to form S1P (Figure 1). As S1P can elicit pro-survival signaling in BC by engaging with five specific G protein-coupled receptors (S1PR1–5) in an autocrine or paracrine manner [26], CDases could be used by cancer cells to their advantage by decreasing the levels of a tumor suppressor (Cer) and increasing the expression of a tumor promoter (S1P) [114].

Like SMases, CDases are classified according to their optimal activity pH [115]. Acid CDase (ASAH1) is a lysosomal enzyme (Figure 2) which preferentially catalyzes the hydrolysis of Cer with C6-C18 fatty acyl chains. Neutral CDase (ASAH2) is a transmembrane glycoprotein found in the plasma membrane and various cellular organelles and uses both Cer and dhCer as a substrate, preferring Cer with C16 and C18 acyl chains. Finally, there are three alkaline CDases (ACER1-3); they are primarily found in the ER and Golgi and preferentially degrade Cer with long-acyl chains (from C20 to C24) [12]. Note that ACER2 selectively catabolizes Cer containing unsaturated forms of C18 and C20 fatty acids (C18:1-CoA and C20:1-CoA) [116].

Among these CDases, the isoform of particular interest in BC seems to be the acid isoform ASAH1. Indeed, higher expressions of ASAH1 detected in ER+ compared to ER- BCs was mainly associated with Luminal A cancers, which are known to have the better prognosis and positive outcomes of all BC subtypes, suggesting the high ASAH1 expression as a useful biomarker for the Luminal phenotype (Table 1) [71]. In addition, the good prognosis of tumors with high ASAH1 was independent of the type of adjuvant treatment in BCs, was positively associated with a reduced frequency of recurrences, and could also be valuable as a prognostic factor for pre-invasive ductal carcinoma in situ (DCIS) [71]. The correlation between ASAH1 and clinical parameters was also more recently investigated in 120 specimens of non-special type invasive ductal carcinoma by Li Yu-hong’s group [72]; these authors found that high ASAH1 expression was correlated with lymph nodes metastasis, but there was no significant difference in ASAH1 expression levels between chemoresistant and chemosensitive groups.

In contrast to these observations, there are some studies that indicated ASAH1 inhibition, which leads to Cer accumulation, as a novel strategy for inducing BC cell death. Indeed, targeting ASAH1 with the small molecular inhibitor ceranib 2 decreased the viability, in a dose- and time-dependent manner, of both MCF-7 and MDA-MB-231 cell lines through the modulation of the mitochondrial membrane potential, but only in MCF-7 cells [73,74], suggesting ceranib 2 as a potent therapeutic agent against both ER+ and ER- BC cell lines. More recently, Vethakanraj et al. [117] have further substantiated the apoptotic activity of ceranib 2 in MCF 7 and MDA MB 231 cell lines, demonstrating that the anticancer effect of ASAH1 inhibitor ceranib-2 in both these tumor cell lines was due to the activation of SAPK/JNK, p38 MAPK apoptotic pathways and the inhibition of the Akt pathway. More importantly, these authors showed that ceranib-2 treatment induced a strong down-regulation of ERα and a docking study predicted a higher binding affinity of ceranib-2 than tamoxifen with ERα in MCF-7 cells [117]. A reduced viability of MCF-7 cells, in a dose-dependent manner, has been also demonstrated by using the Cer analogue D-erythro-MAPP and its nanoparticle formulation [75], supporting ASAH1 as a promising druggable target.

### 5.4. Sphingosine-1-Phosphate Lyase (S1PL) and Sphingosine-1-Phosphate Phosphatases (S1PP and LPP)

Sphingosine-1-phosphate lyase (S1PL) and sphingosine-1-phosphate phosphatase (S1PP) are responsible for the clearance of the anti-apoptotic and pro-survival S1P [12]. In particular, S1P can be irreversibly cleaved by S1PL1 to ethanolamine-1-phosphate and trans-2-hexadecenal, representing the metabolic step of the exit of SL metabolism (Figure 1). Alternatively, S1P can be dephosphorylated to Sph, which in turn can be recycled to generate Cer again through the salvage pathway, by two S1P-specific phosphatases (S1PP1/2), but also by three non-specific lipid phosphatases (LPP1-3) (Figure 1) [118,119,120].

Despite its strategic role in S1P removal, to date very little attention has been paid to S1PL in BCs. Only recently, Engel et al. [76] provided evidence of very low S1PL1 protein levels in BC tissues, regardless of their subtype, compared with corresponding healthy tissues, and of a stringent correlation with a poorer overall and relapse-free survival (Table 1). Furthermore, lower S1PL1 protein expression, but not of the corresponding mRNA, was also observed in the Luminal A MCF-7 cells and in the most aggressive triple-negative MDA-MB-231 and BT-20 cells, compared with two non-tumorigenic, epithelial breast cell lines (i.e., MCF-12A and MCF-10A). However, these authors also observed that S1PL1, typically localized in the ER, where it only has access to cytosolically produced S1P as their catalytic site faces the cytosolic face of the ER [12], was also markedly detectable in the plasma membrane of non-tumorigenic breast cells (Figure 2). On the contrary, cell surface signals of S1PL1 were much lower in MCF-7 cells and absent in BT-20 and MDA-MB-231 cells. As the general S1PL1 downregulation and the loss of the plasma membrane expression resulted in S1P-dependent stimulation of migration in MCF-7 and BT-20 cells, whereas S1PL1 overexpression restored the migration behavior to control levels, it has been suggested that low S1PL1 expression levels and especially the loss of S1PL1 plasma membrane distribution could have been a potential prognostic marker and a viable target for therapeutic interventions [76].

Similarly, studies on the role of the dephosphorylation of S1P by S1PP or LPP enzymes in BCs are limited. Particularly up-and-coming are the recent findings described by Nema and Kumar [77], who have reported a significant positive association between lower mRNA expression levels of S1PP1 and LPP3 in BC specimens, compared to normal tissues, and relapse-free survival and overall survival in patients with BCs (Table 1). Moreover, these authors have found that both S1PP1 and LPP3 showed a prognostic value highly dependent on BC intrinsic subtypes, pathological grades, and lymph node status; in particular, SGPP1 and LPP3 expression decreased in primary tumors compared to normal breast tissues, with major reduction in TNBC subtypes and stage IV patients [77]. Furthermore, whereas S1PP1 had a high predictive value in the HER2+ BCs and TNBCs, LPP3 had a high predictive value in Luminal A BCs. Both enzymes also possessed a high predictive value for responses to systematic therapy in invasive BC patients, especially in the HER2+ and TNBC subtypes. Finally, since the active site of LLP3 faces the extracellular matrix, the low levels of this phosphatase could decrease the degradation of S1P present in the extracellular environment to hinder the recruitment of immune cells, such as DCs, CD4+, and CD8+ T cells into the tumor stroma, resulting in the blockage of cancer cell removal and a subsequent poor prognosis [77]. The mechanisms by which S1PP1 and LPP3 are decreased in the tumors of BC patients are not fully understood. However, whereas a decrease in the mRNA expression of LPP3 in BCs was found to be associated with the promoter hypermethylation, S1PP1 promoter hypermethylation does not seem to be involved in the repression of its gene expression [77].

In addition, although the potential prognostic markers identified by Nema and Kumar in the prediction of the effectiveness of systemic therapy in TNBC patients (particularly S1PP1) need to be validated further, all these new findings support that the decreased expression of S1P-catabolizing enzymes, the accumulation of S1P within cells and its significantly increased secretion into the extracellular milieu may be cause of the resistance of MCF-7 cells to TNF-α and daunorubicin [78,79].

## 6. Future Perspectives

Current evidence strongly sustains the assumption that alterations in the sphingolipidome play crucial roles in cancer biology and therapeutics [11,22,121]. Indeed, significant advances have been made in both molecular and analytical tools to study SL metabolism over the years, and the deregulation of specific SL species, as well as of their metabolizing enzymes, has been found in both BC cell lines and patient tissues, although the functional association of specific SL metabolites and/or enzymes with BC pathogenesis and drug response is still only partly understood.

Cer and S1P are currently believed to be the most important bioactive SLs regulating key cellular functions in transformed breast cells, as well as in their response to therapy. Indeed, it is generally believed that an increased S1P/Cer ratio contributes to BC progression through the upregulation of tumor cell survival, proliferation, migration, and invasion, and it can simultaneously contribute to the prevention and reduction of sensitivity of BCs to drug-induced apoptosis [24,25,26]. However, current and growing findings highlight that the dysregulation of SL metabolism in BC is much more complex than originally imagined, and that the ultimate fate of the cell arises from a complex and dynamic balance of many other SL metabolites which, in turn, may elicit mitogenic effects or contribute to the regulation of cell death. Indeed, as summarized in Figure 1 and Figure 2, SL metabolism consists of a highly dynamic network of interconnected anabolic and catabolic enzymatic activities through which the pro-apoptotic Cer, the central hub of SL metabolism, can accumulate by the activation of at least three different pathways (synthesis de novo, SM hydrolysis and salvage pathway), and also its accumulation can be neutralized by the upregulation of SMS or CerK or GCS or CDase which, producing SM, C1P, GlcCer and Sph, respectively, promote cell survival, proliferation, migration, invasion, metastasis development, alteration in immune cell recruitment into the tumor environment, and, last but not least, resistance to apoptosis and impaired therapeutic response with different mechanisms to a different extent. Furthermore, since one stimulus can lead to the simultaneous regulation of more than one enzyme (both anabolic and catabolic) of SL metabolism, a single stimulus will be able to elicit diverse responses leading to the multiple BC phenotypes and variable therapy outcomes.

Therefore, although the two-dimensional model referred to as the ‘sphingolipid rheostat’ may well reproduce the balance between S1P and Cer, a more inclusive ‘sphingodynamic’ model that accounts for the complexity of SL metabolism and for changes within a greater number of bioactive SLs should be considered. Dissection of each component of SL metabolism, including dhCer and other species with varying sphingoid backbones and/or N-linked acyl-CoA, as well as intracellular SL transporters and neutral GSLs, is certainly important, but the interplay between SL enzymes, their substrates and their products is often more complex than the static lipid levels reveal. Therefore, to add context to the alterations in cellular SL levels in BC biology and the response to specific drugs, it is essential that eventual changes in SL species are not evaluated individually, focusing on just a few isolated species, but within the context of the whole sphingolipidome because it is the impaired combinatorial anabolic and catabolic metabolism of multiple SL species that should be investigated to determine whether therapeutic targeting of the SL network is to be effective. A recent example of the reasonableness of this approach is offered by the study by Snider’s group [80], who, using d17-dhSph as a probe, monitored the effects of cytostatic and cytotoxic doses of Dox on SL metabolic flux, and gave a spatial context to Sph generated by CDase in response to the cytostatic Dox dose.

However, the application of this approach could be limited by the fact that the same lipid species can be generated not only by different metabolic pathways, but also by different enzymatic isoforms that, localizing in different subcellular compartments, may have different functions and/or different mechanisms of action in mediating the survival or death of BC cells, as previously described in the case of SMS1 and SMS2 [52,53]. In addition, as occurring for all enzymes, the activity of those involved in SL metabolism may also be regulated at the transcriptional and/or post-translational level. Unfortunately, to date, most studies on BC cells and tissues define SL enzymatic changes compared to control samples only by measuring the corresponding mRNA levels, eventually induced or suppressed by transfection with vectors containing cDNA o siRNA, and rarely correlate data with the levels of the corresponding protein product.

Finally, since an essential requirement for having a personalized and more effective treatment of BC patients with drugs that interfere with the SL metabolism is the knowledge of sphingolipidome in tumors, deciphering the sphingolipidome in BC cells and patients’ tissues in detail, before and after specific drug treatment, can certainly help to identify new diagnostic and prognostic SL-based biomarker(s) so as to better typify the different subtypes of BCs and to be useful for developing novel therapeutic and prevention strategies against BCs. However, the application of potential SL-based biomarker(s) could be hampered by an insufficient understanding of their biological significance and can be further complicated by the fact that most of the currently available data are derived from whole tumor tissue, which may contain numerous distinct cell types.

Until now, the identification of potential biomarkers has only been possible when the tumor is resected and analyzed in vitro, an approach not always possible. Therefore, if on the one hand the identification of new specific biomarkers is very important to determine the SL status in tumor tissues, on the other hand it would be more useful for clinicians to know the SP status of the tumors in vivo. Evaluating whether SL plasma levels can be predictive determinants for the SL status in tumors was the main objective of the metabolomic approach applied in two studies. Indeed, Miolo et al. [122] identified circulating spermidine and tryptophan as potential markers associated with the complete pathological response to trastuzumab-paclitaxel neoadjuvant therapy in HER2+ BC.More interestingly, Monzen S. et al. [123] recently reported not only a parallel expression intensity of HER2 and nSMase2, possibly connected to cell communication by extracellular vesicles (EVs) [124], but also that the serum concentration of four SM species [SM 24:1, SM C26:0, hydroxySM C16:1 and hydroxySM C24:1] were inversely related to the accumulation of nSMase2 in HER2+ cancer tissues from a patient who exhibited complete therapeutic resistance to monoclonal-anti-HER2 antibody therapy, chemotherapy and radiotherapy after mastectomy. Clearly, further and detailed analyses are needed on a larger number of patients.

**Figure 3:**
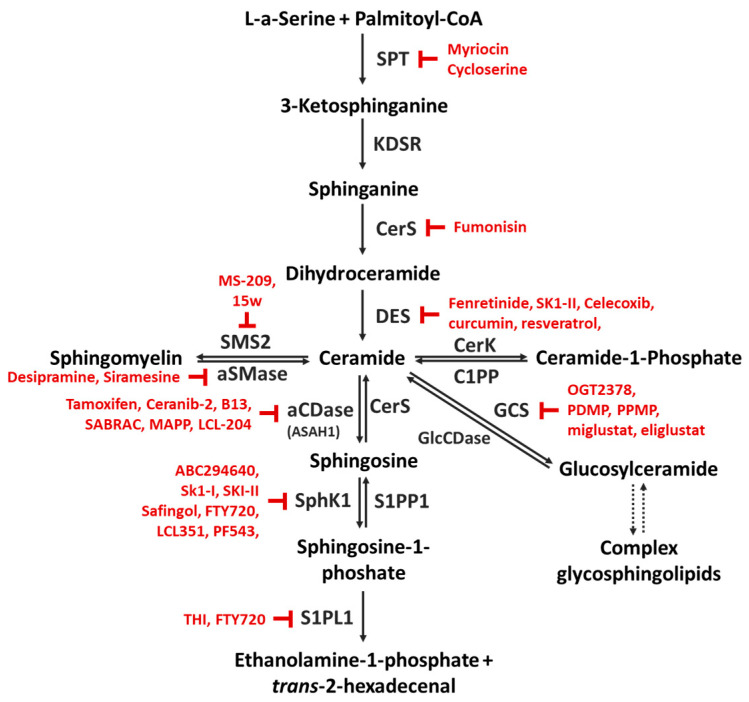
Some chemical inhibitors used to target sphingolipid metabolism. Abbreviations: aCDase, acid ceramidase; CerK, ceramide kinase; CerS, (dihydro)ceramide synthase; C1PP, ceramide-1-phosphate phosphatase; DES, dihydroceramide desaturase; GCS, glucosylceramide synthase; GlcCDase, glucosylceramidase; KDSR, 3-ketosphinganine reductase; PDMP, 1-phenyl-2-decanoylamino-3-morpholino-1-propanol; PPMP, 1-phenyl-2-palmitoylamino-3-morpholino-1- propanol; aSMase, acid sphingomyelinase; SMS2, sphingomyelin synthase 2; SphK1, sphingosine kinase 1; S1PL1, sphingosine-1-phosphate lyase; S1PP1, sphingosine 1-phosphate phosphatase; SPT, L-serine palmitoyltransferase.

Although there are still open questions about how BC patients could be identified as target subjects for SL therapy, there is a great deal of knowledge about the involvement of Cer with specific acyl chain length and of other SL metabolites in BC progression and in the development of chemoresistance, and current data indicate that targeting the SL pathways as a new option in BC therapy has already moved forward. Clearly, in order to improve and broaden BC treatment options further, detailed investigations into the specific effects of distinct SL species and into the molecular mechanisms leading to their interconnected changes in BC development and drug-resistance are needed. Furthermore, the present overview of the alterations of intracellular SL concentrations and of deregulated enzymes in BCs may help to improve the identification of major points of SL metabolism that could be targeted by the use of various chemical inhibitors (some of those already available are represented in Figure 3), siRNAs, antibodies or a direct treatment with Cer analogs (i.e., encapsulated in nanoliposomes) or additional therapeutic approaches so as to lay the basis for transferring all these efforts and knowledge into clinical practice.

## 7. Conclusions

The aim of this review was to provide a deep and comprehensive discussion on the molecular peculiarities of the SL metabolism in BC and its impact on the tumor growth, the success of conventional anti-cancer therapies, the onset of drug resistance and tumor relapse.

Collectively, current knowledge here summarized strongly supports the importance of SL metabolism as an innovative hallmark to discriminate BC subclasses and provides basic information that might lead to the engineering of personalized and innovative pharmacological adjuvant strategies. Nevertheless, it is our opinion that the possibility of designing clinical studies based on the mechanisms described would be too speculative at the moment because of both the wide histological and molecular heterogeneity of BC and the higher complex of SL metabolism than that originally imagined. In addition, while summarizing the information available to date, it emerged that at least three prerequisites would have to be fulfilled before transferring all the efforts and knowledge to clinical practice: 1. to develop sphingolipidomic strategies capable of providing a complete SL profile of each BC subtype; 2. to test the actual antitumoral efficacy and adverse effects of the compounds and drugs that interfere with SL metabolism in preclinical studies; 3. to identify patients that might actually benefit from a specific SL-targeted therapy, so as to perform clinical trials to dissect these compounds thoroughly.

## Figures and Tables

**Table 1:** Sphingolipid enzymes potentially involved in breast cancer growth and response to therapies.

Sphingolipid Enzyme	Main Functions in BC Tissues and Cells	References
Serine-palmitoyl-CoA transferase (SPT)	Alternative sphingoid bases induce apoptosis in MCF-7, in MDA-MB-231 and MCF-7/Adr cell lines or block the endogenous synthesis of Cer	[14,32,33,34,35]
3-ketosphinganine reductase (KDSR)	↑ KDSR in MDA-MB-231 cells induces toxic accumulation of 3KDS	[36]
Ceramide synthase (CerS)	↑ CerS2 is positively correlated with longer disease-free and overall survival of patients, reverse relationship with tumor progression, lymph node metastasis and HER2 expression	[37,38]
	↑ CerS2 inhibits tumor growth in nude mice, and migration and invasion of MDA-MB-231 cells	[37]
	↓ CerS2 predicts chemoresistance in MCF-7/Adr cells and increases MCF-7 cell migration and invasion	[37,39]
	↑ CerS2 inhibits MDA-MB-231 cell migration and invasivity	[39]
	↑ CerS6 is related to poor prognosis, lymph node involvement and metastasis in patients	[40,41,42]
	↑ CerS6 in ER+ BCs with respect to ER- BCs	[40,43,44]
	↑ CerS6 in Luminal BCs with respect to TNBCs	[45]
	↑ CerS6 inhibits cell migration of the mesenchymal human MDA-MB-231 and MDA-MB-468 cells	[45]
	↓ CerS6 increases cell migration in the epithelial human MCF-7 and T47D cells	[45]
	↓ CerS6 acts as up-stream effector of the loss of focal adhesion protein and plasma membrane permeabilization in TNF-α treated MCF-7 cells	[46]
	↓ CerS6 improves pemetrexed-induced lethal autophagy	[47]
Dihydroceramide desaturase 1 (DES1)	↑ DES1 is found in a subset of HER2+ tumors, associated with aggressive and metastatic phenotype and worse survival outcomes of the patients	[48]
	↑ DES1 is a potential biomarker of aggressive and metastasis-prone HER2+ BC	[48]
	↓ DES1 by 4-HPR inhibitor, alone or in combination with tamoxifen, accumulates dihydroceramides, prevents relapses in patients and promotes high, durable anticancer effects in MCF-7 and MDA-MB-231	[49,50]
	↓ DES1 by 4-HPR inhibitor associates with HER2-driven anchorage-independent survival in HER2+ SKBR3 cell lines	[48]
Sphingomyelin synthase (SMS)	↓ (SMS2 mRNA correlates with less BC aggressivity in patients	[42]
	↑ (SMS2 expression is higher in Luminal BCs than TNBCs and associates with poor prognosis	[51]
	↑ (SMS2 mRNA associates with BC metastasis in patients	[52]
	↑ (SMS2 promotes cancer cell proliferation, migration and invasiveness in MCF-7 and MDA-MB-231 cells and in vivo by enhancing EMT mediated by TGF-β/Smad pathway	[52]
	↑ (SMS2 facilitates M2-macrophage polarization in TNBCs and their infiltration in the tumor stroma in a mouse model of TNBCs	[52]
	↑ (SMS1 inhibits EMT mediated by TGF-β/Smad pathway and reduces MDA-MB-231 cell migration and invasion induced by TGF-β1	[53,54]
Sphingomyelinase (SMase)	↑ (SMase2 correlates with less BC aggressivity in patients	[42]
	↑ (SMase2 mRNA is found in Dox-resistant MCF-7 cells compared to Dox-sensitive parental cells	[54,55]
Ceramide kinase (CerK)	↑ (CerK correlates with aggressivity tumors and poor clinical outcomes following adjuvant or neoadjuvant therapy	[56,57]
	↑ (CerK mRNA is in ER- BCs patients, compared to those ER+ BC, and associates with worst prognosis, shorter survival, and increased risk of recurrence, likely due to the rapid upregulation of CerK following chemotherapy	[58,59]
	↑ (CerK promotes migration and invasion of metastatic MDA-MB-231 cells	[60,61]
	↓ (CerK by NVP-231 inhibitor reduces MCF-7 cell proliferation by inducing apoptosis	[57,60]
Glucosylceramide synthase (GCS)	↑ (GCS increases proliferation, tumor progression and poor prognosis in patients	[62]
	↑ (GCS is a potential useful biomarker of BC progression	[62,63]
	↑ (GCS is in MCF/Adr-resistant cells mediated by ABCB1 protein	[64,65,66,67,68,69]
	↓ (GCS by PDMP inhibitor restores sensitivity to vinblastine treatment and stimulates autophagy in MCF-7 cells	[31,70]
Acid Ceramidase (aCDase, ASAH1)	↑ (ASAH1 mRNA is in ER+ BCs compared to ER- BCs	[71]
	↑ (ASAH1 is a potential useful biomarker in Luminal A	[71]
	↑ (ASAH1 is a possible prognostic factor for pre-invasive ductal carcinoma in situ and positively correlates with a reduced frequency of recurrences	[71,72]
	↓ (ASAH1 by ceranib 2 inhibitor decreases viability of MCF-7 and MDA-MB-231 cells	[73,74]
	↓ (ASAH1 by D-erythro-MAPP inhibitor decreases viability of MCF-7 cells	[75]
Sphingosine-1-phosphate lyase (S1PL)	↓ (S1PL in BC tissues, regardless of their subtype, and correlates with a poorer overall and relapse-free survival in patients	[76]
	↓ (S1PL is in Luminal A MCF-7 cells and triple negative MDA-MB-231 and BT-20 cells	[76]
	↓ (S1PL expression in plasma membrane has a potential prognostic value of cell migration and invasion properties	[76]
Sphingosine-1-phosphate phosphatases (S1PP) Lipid phosphatases 3 (LPP3)	↓ (S1PP and ↓ (LPP3 correlate with an overall and relapse-free survival in patients	[77]
	↓ (S1PP and ↓ (LPP3 have a prognostic value highly dependent on BC intrinsic subtypes, pathological grades, and lymph node status; S1PP has high predictive value in TNBC subtypes and stage IV patients, and LPP3 in Luminal A	[77]
	↓ (S1PP and LPP3 show a high predictive value for response to systematic therapy in invasive BC patients, especially in the HER2+ and TNBC subtypes	[77]
	↓ (S1PP and LPP3 may hinder the recruitment of immune cells, especially DCs, into the tumor environment	[77]
	↓ (S1PP is implicated in resistance of MCF/7 cells to TNF-α and daunorubicin	[78,79]

## Data Availability

Not applicable.
